# Virtual Reality: Challenges and Perspectives in Mental Health

**DOI:** 10.31083/AP47536

**Published:** 2025-10-21

**Authors:** Sergio Machado, Flávia Paes, Rafael Ferreira-Garcia, Lucio Lage Gonçalves, Mauro Giovanni Carta, José Carlos Appolinario, Antonio Egidio Nardi

**Affiliations:** ^1^Virtual Reality in Mental Health Laboratory (RVSM), Institute of Psychiatry (IPUB), Federal University of Rio de Janeiro (UFRJ), 21941-908 Rio de Janeiro, Brazil; ^2^LABPR – Panic and Respiration Laboratory (LABPR), Institute of Psychiatry (IPUB), Federal University of Rio de Janeiro (UFRJ), 21941-908 Rio de Janeiro, Brazil; ^3^Center of Neuroscience, Neurodiversity Institute, 26325-020 Queimados, RJ, Brazil; ^4^Department of Medical Sciences and Public Health, University of Cagliari, 09124 Cagliari, Italy; ^5^Obesity and Eating Disorder Group (GOTA), Institute of Psychiatry (IPUB), Federal University of Rio de Janeiro (UFRJ), 21941-908 Rio de Janeiro, Brazil

**Keywords:** virtual reality, psychiatric disorder, virtual reality exposure therapy

## Abstract

Virtual reality (VR) is emerging as a revolutionary tool in mental health, offering new approaches to treating psychiatric disorders. Its ability to create immersive environments allows patients to safely address psychological challenges. This article reviews current applications of VR in mental health, its limitations, and future directions for research. VR has been applied to the treatment of phobias, anxiety disorders, post-traumatic stress disorder (PTSD), and eating disorders. Virtual reality exposure therapy (VRET) facilitates desensitization by allowing gradual exposures to feared stimuli, demonstrating efficacy in reducing symptoms of anxiety and social phobias. In relation to PTSD, VR helps patients to process traumatic memories in a controlled environment, proving to be a promising alternative for those who do not respond to traditional treatments. While VR offers significant benefits, such as personalization of interventions and real-time data collection, it faces methodological and accessibility challenges. The lack of rigorous research and the need for specialized equipment limit its implementation. Future research should focus on large-scale studies and the integration of VR with technologies such as artificial intelligence and biofeedback, which can improve the personalization of treatment. In conclusion, VR has transformative potential in psychiatry but, to be fully effective, it is essential to overcome existing challenges and increase its accessibility, promoting responsible and ethical use.

## Main Points

1. Therapeutic Potential of Virtual Reality (VR)

VR facilitates immersive and controlled exposure therapy for various conditions such as phobias, post-traumatic stress disorder (PTSD), and anxiety disorders, enabling patients to safely confront their triggers while practicing coping mechanisms. The ability to customize and repeat sessions enhances the effectiveness of treatment.

2. Clinical Applications

VR has demonstrated effectiveness in addressing specific disorders (for instance, social anxiety, psychosis, and eating disorders) through customized simulations. It has the potential to decrease reliance on therapists, enhance accessibility, and complement therapies such as cognitive behavioral therapy (CBT).

3. Advantages Over Traditional Methods

VR provides distinct advantages, including real-time biofeedback, virtual embodiment for addressing body image concerns, and scalable remote therapy options. Its immersive characteristics boost patient engagement and allow for scenarios that are impractical in real life (such as trauma reenactment).

4. Challenges and Risks

Challenges include methodological deficiencies in research, high expenses, and technical constraints (such as cybersickness). Side effects, including temporary spikes in anxiety or feelings of depersonalization, necessitate careful clinical supervision.

5. Future Directions

The integration of artificial intelligence (AI) and biofeedback could further personalize therapeutic interventions. To validate the long-term effectiveness and ethical application of VR in mental health care, large-scale studies, standardized protocols, and clinician training are essential.

## 1. Introduction

Virtual reality (VR) is emerging as a revolutionary tool in mental health, providing new approaches to the treatment of psychiatric disorders [1]. VR’s ability to create immersive environments offers significant therapeutic potential, allowing patients to face and overcome psychological challenges in a safe and controlled space [2].

VR has remarkable potential to assist individuals in overcoming mental health challenges, particularly if a strong sense of presence is established in troubling situations. Difficulties in interacting with the world are central to many mental health issues, such as experiencing intense anxiety around spiders in arachnophobia, having severe flashbacks triggered by reminders of past trauma in post-traumatic stress disorder (PTSD), fearing attacks from others in persecutory delusions, or struggling to resist the urge to drink in alcohol abuse disorders [3]. Therefore, recovery involves changing thoughts, reactions, and behaviors in these contexts. The most effective interventions are those that facilitate such changes in real-life situations [4].

VR enables individuals to engage in immersive simulations of complex scenarios, providing them with guidance on suitable responses based on the most effective theoretical frameworks for their specific conditions. These simulations can be tailored in terms of difficulty and repeated as necessary to achieve the desired level of learning [5]. Rare real-life situations can be effortlessly recreated with a simple command. A notable benefit of VR is that users recognize the virtual setting as artificial; however, their psychological and physiological reactions are akin to those experienced in reality. This phenomenon often leads individuals to be more inclined to face challenging situations in a virtual context than in actual life, thereby allowing them to explore new therapeutic approaches. The skills acquired in these simulations can subsequently be applied to real-world scenarios [6].

For certain disorders, it may even be possible to reduce or eliminate the need for therapist involvement, while for others, the demand on skilled therapists could be significantly lessened. Consequently, VR has the potential to improve access to effective psychological therapies and may emerge as a preferred therapeutic modality, shifting from traditional methods to innovative headset-based experiences [5].

With VR, individuals can immerse themselves in simulations of challenging scenarios and receive guidance on appropriate responses, rooted in the best theoretical insights for their specific disorder. The difficulty level of these simulations can be adjusted and repeated until the desired learning occurs [5]. Difficult-to-find real-life situations can be recreated easily at the push of a button. A significant advantage of VR is that users understand that the virtual environment is not real, yet their minds and bodies respond as if it was; consequently, individuals are often more willing to confront difficult scenarios in VR than in real life, allowing them to experiment with new therapeutic strategies. This learning can then transfer to real-world situations [6].

For certain disorders, it might even be feasible to eliminate the need for therapist involvement, while for others, the time required from skilled therapists could be significantly reduced. Thus, VR could enhance access to effective psychological treatments and may become the preferred method for therapy: moving away from the traditional couch and embracing the headset [5].

The interest of the scientific and professional community in VR as a valuable therapeutic tool has been demonstrated by the increasing number of scientific publications on the subject in recent years. Therefore, this review article aims to explore the current applications of VR in mental health, discuss its limitations and challenges, and suggest future directions for research and clinical practice.

## 2. Concepts and Principles of Virtual Reality

VR can be described as a user-computer interface approach that involves the real-time simulation of an environment, scenario, or activity, allowing the user to interact with it through multiple sensory channels. VR combines computer-generated imagery, sound, and various sensory inputs to create an interactive digital environment [6]. Unlike traditional computer displays, this virtual setting is experienced through a head-mounted display (HMD), which typically consists of a helmet or goggles featuring dual small screens and stereo headphones. Users can navigate and interact within this virtual space using motion-tracking devices linked to the HMD, and sometimes to their hands or feet, allowing the system to modify the visual perspective in response to the user’s movements [5]. A successful VR experience fosters a sense of “presence”, making users feel as if they are physically located within the virtual environment. This immersion is facilitated by minimizing external real-world distractions, ensuring that only the computer-generated surroundings are perceived. Furthermore, some advanced VR systems incorporate haptic feedback through devices such as data gloves, which enhance the experience by engaging multiple senses, including sound, touch, and even smell, thereby intensifying the sense of realism [6].

VR technology has experienced significant advancements since its initial use in the field of mental health. Modern computers operate at much higher speeds and the quality of head-mounted displays has seen considerable enhancement. Perhaps most notably, the reduction in technology costs has led to the creation of a wider array of applications [5, 6]. The core emphasis of VR lies in interaction and it can be anticipated that as the technology progresses and facilitates more extensive interaction, its efficacy as a therapeutic instrument will likewise improve. Nevertheless, “low-tech” environments, such as those based on computer games, have already demonstrated positive outcomes. Participants are influenced by these environments, despite their awareness of their virtual nature [7].

A number of applications of VR have been developed for use in mental health, including treatments for specific phobias, such as fear of flying [8, 9], fear of heights [10, 11], fear of spiders [12, 13], and fear of cockroaches [14]. The first study of VR technology in mental health was by Rothbaum *et al*. [15] that carried out the initial research in psychiatry to explore the effectiveness of VR in treating acrophobia among college students. In this study, 20 college students with acrophobia were randomly divided into VR graded exposure treatment (n = 12) or to a waiting-list comparison group (n = 8). Sessions were held individually over a period of 8 weeks. Outcomes were evaluated using assessments of anxiety, avoidance, attitudes, and distress related to exposure to heights, both before and after the treatment. Significant differences were observed between the students who underwent the VR therapy (N = 10) and those who were on the waiting list (N = 7) across all measures. The treatment group showed notable improvements after 8 weeks, while the comparison group remained unchanged. These findings indicated that VR successfully diminished their fear of heights.

Various applications of VR have been created to address conditions such as post-traumatic stress disorder [16], attention deficit disorder in children [17], and anxiety [18]. Additionally, VR technologies have been designed to support the cognitive evaluation and rehabilitation of individuals suffering from traumatic brain injuries, strokes [19], dementia [20], and schizophrenia [21, 22]. The potential advantages of therapy utilizing VR are evident, particularly in the context of anxiety disorders. As with traditional approaches, VR therapy for these conditions relies on the principle of exposure. In certain cases, direct exposure may be impractical, challenging, or even hazardous (as seen with driving phobias), while in other instances, the financial burden may be excessive (as with flying phobias). In such scenarios, VR presents a safe and economically viable alternative.

Virtual reality exposure therapy (VRET) shares similarities with imaginal exposure, as it takes place in a controlled setting, typically within the therapist’s office. This method allows for the elicitation of fear responses without the necessity of confronting real-world scenarios, thereby ensuring confidentiality [6]. Patients may find VR exposure to be a safer alternative to traditional in-person exposure, as they have the ability to terminate the experience at any moment. This sense of control over the VR environment can enhance patients’ self-efficacy [5]. Additionally, a less intimidating setting for those with phobias may motivate more individuals to pursue treatment and decrease the likelihood of dropout. Most VR environments offer flexibility, enabling therapists to customize the experience according to each patient’s specific fear hierarchy [7].

VR can be employed to simulate experiences that individuals cannot be re-experienced in person, such as combat scenarios or the events surrounding the attack on the World Trade Center. This technology is particularly advantageous for individuals suffering from PTSD [23]. VRET offers a viable alternative to traditional imaginal exposure methods, as it removes the necessity for patients to depend on their internal imagery or visualization skills. Additionally, while therapists lack control over the specific images that patients may visualize during imaginal exposure, the virtual environment allows for precise regulation of the stimuli presented to the patient [4]. Similar to its application with phobic patients, VR-based exposure therapy may prove especially effective for those with PTSD, as their tendencies to avoid certain situations and their resistance to treatment can impede the therapeutic process [23].

## 3. Therapeutic Applications of Virtual Reality in Mental Health

VR has been applied in the treatment of a variety of psychiatric conditions, such as phobias, anxiety disorders, eating disorders, PTSD, and psychosis [3]. In the treatment of phobias, for example, VR allows controlled and gradual exposures to feared stimuli, an essential component for desensitization. This technique is known as VRET, which is effective in reducing symptoms of anxiety [6] and social phobia [4]. The ability to replicate stimuli that trigger reactions in a controlled environment is one of the main benefits of VRET, enabling a gradual and safe therapeutic approach [6].

VRET has been widely researched and applied in anxiety disorders. Studies have demonstrated the effectiveness of VR in creating exposure scenarios that simulate real-world situations, with a reduction in anxiety symptoms [6]. VRET was found to be more effective than wait list individuals in reducing anxiety symptoms. VRET demonstrated similar effects to other interventions in post-intervention and follow-up assessments, with a greater effect for participants with symptomatic social anxiety when combined with cognitive behavioral therapy (CBT) compared with its counterpart. Overall, VRET may provide supplemental therapy to improve anxiety symptoms [6]. Additional high-quality, large-scale trials with long-term follow-up are needed.

In the context of PTSD, VRET can effectively simulate traumatic experiences within a secure setting, enabling patients to confront and process their traumatic memories in a structured way, thereby promoting emotional processing and recovery [16, 24]. A study conducted by Eshuis *et al*. [23] demonstrated that VRET outperformed control groups and exhibited efficacy comparable with other forms of psychotherapy. Nonetheless, the findings revealed significant variability, attributed to the limited number of studies and the diverse VR methodologies employed. The analysis encompassed eleven studies involving 438 participants, most of which were of subpar quality and yielded inconsistent results. The data indicated that VRET was more effective than wait-list control (standardized mean difference –0.64 (95% CI –1.05 to –0.22)), while no notable differences were observed between VRET and active treatment conditions (standardized mean difference –0.25 (95% CI –0.77 to 0.27)). VRET presents a promising alternative for treating PTSD, particularly for patients who have not benefited from previous interventions. Future investigations should prioritize high-quality randomized clinical trials (RCTs) that include assessments of side effects and adverse events, with a sufficient participant pool. Conversely, research by Heo and Park [16] indicated that graded VRET produced a significantly greater effect size for PTSD symptoms (g = 1.100, *p* = 0.001) in comparison with control groups. However, no significant difference was observed between conventional VRET and control groups regarding PTSD symptoms (g = –0.279, *p* = 0.970). These results underscore the effectiveness of graded VRET for alleviating PTSD symptoms, highlighting the potential of immersive treatments in this domain.

In eating disorders, VRET has been explored as a promising approach to address body image distortion and dysfunctional eating behaviors [24]. Studies such as the one by Riva *et al*. [24] focused on VRET as a novel method for addressing eating disorders, including anorexia and bulimia. VRET creates an immersive setting that assists patients in confronting and managing their anxiety associated with body image, eating habits, and related behaviors. Research indicates that VRET can effectively expose individuals to scenarios that provoke maladaptive eating patterns, enabling them to practice coping strategies within a secure environment. Additionally, this technology can be customized to meet the specific needs of each patient, presenting tailored scenarios that tackle their distinct challenges. While VRET should not replace traditional therapies such as CBT, it can serve as a supplementary resource, enhancing the overall effectiveness of treatment interventions. Nonetheless, there are obstacles to the implementation of VRET, including the necessity for specialized equipment and some patients’ reluctance to embrace new technologies. We emphasize the need for further research to evaluate the long-term efficacy of VRET across various populations and to investigate its potential applications for other eating disorders. In summary, the integration of VRET into the treatment of eating disorders holds significant promise, offering innovative approaches for patient rehabilitation, although it necessitates additional research and development. Additionally, de Carvalho *et al*.’s study [25] suggests that VRET has an important utility in eating disorders. In this systematic review, 19 articles were selected (9 for assessment and 10 for treatment). The existing evidence suggests that the application of VRET in evaluating these conditions has demonstrated potential in revealing: (1) the patients’ perceptions of their body image; and (2) particular settings or food types that could initiate the binge-purge cycle. Research utilizing VR-based environments in conjunction with cognitive-behavioral strategies has indicated its possible effectiveness in enhancing motivation for change, boosting self-esteem, addressing body image issues, and decreasing binge-eating and purging behaviors.

Finally, psychosis has also been the target of VRET. The review of Rus-Calafell *et al*. [26] shows how VR can be applied to simulate psychotic experiences, allowing for a better understanding of symptoms and more effective training in social skills. Fifty studies were included, and the results indicate that VRET is generally well accepted by patients and professionals, providing a safe environment for exposure to situations that may trigger symptoms. Furthermore, VRET has demonstrated efficacy, such as the rehabilitation of social skills and the reduction of psychotic symptoms. Despite its benefits, the study also highlights the need for more research to better understand the long-term efficacy and feasibility of VRET in different clinical contexts. In summary, VR has emerged as a promising tool in the assessment and treatment of psychosis, although more studies are needed to consolidate its applications.

The mirror neuron system is a possible neurological mechanism responsible for the improvements when using VR. Within this context, a study using immersive VR in combination with imaging techniques showed that individuals without bipolar disorder, when faced with emotional tasks that require cognitive processing and responses, show activation of the right anterior cingulate cortex, insular cortex, and inferior frontal cortex, which compose the “mirror neuron system”, while individuals with bipolar disorder show reduced activity in these brain areas [27]. The “mirror neuron system” is essential for understanding the actions and behavior of others but is also essential for becoming aware of one’s own emotions and behavioral patterns [28]. Therefore, this system is implicated in complex neurocognitive functions, such as social cognition, theory of mind, empathy, and language [29]. In this context, it seems that VR exercises can stimulate the mirror neuron system through a “bombardment” of similar conditions in the real world.

VR offers unparalleled advantages in the development of treatment strategies for mental health disorders, enabling methods that were previously unattainable or impractical with traditional therapeutic approaches [6]. One of the key benefits of VR is its ability to provide controlled and safe environments for therapeutic interventions. In conventional exposure therapy, individuals dealing with phobias or PTSD often encounter real-world situations that can be unpredictable, dangerous, or difficult to replicate [7]. VR addresses these issues by allowing therapists to simulate specific scenarios—such as a crowded social event for someone with social anxiety or a combat environment for a veteran with PTSD—within a completely secure and controlled setting. This level of control enables patients to confront their fears gradually and at a pace that is comfortable for them, which is crucial for successful treatment. Research has demonstrated the efficacy of VRET for conditions like PTSD and phobias, showing significant reductions in symptom severity [30, 31].

Another notable benefit of VR lies in its capacity for customization and replication. Therapists have the ability to design virtual environments tailored to the unique needs and experiences of each patient [6]. For instance, a patient with a fear of flying can be immersed in a virtual airplane cabin that closely mimics the actual conditions of a flight, encompassing takeoff, turbulence, and landing. This level of personalization is unattainable with traditional methods, which often rely on generic or improvised scenarios [2]. Moreover, VR allows for the consistent reproduction of these scenarios as needed, ensuring uniformity across therapy sessions. This consistency is particularly vital for conditions such as PTSD, where patients must confront specific traumatic experiences in a controlled environment to effectively process their emotions. Research has shown that customized VR environments enhance treatment outcomes by increasing patient engagement and relevance [2].

Additionally, the immersive and engaging qualities of VR set it apart from conventional therapeutic methods. VR creates a strong sense of presence, enabling patients to feel as though they are truly “inside” the virtual environment. This immersion enhances the effectiveness of therapeutic techniques by making the experience more authentic and impactful [6]. For example, individuals with social anxiety can practice interactions with virtual avatars in a realistic social setting, which often feels more genuine than traditional role-playing with a therapist [2]. Similarly, VR can be employed to develop serene, immersive environments for mindfulness and relaxation exercises, allowing patients to achieve a deeper state of calm than they might through standard methods. A study by Maples-Keller *et al*. [3] indicated that immersion in VR environments significantly boosts engagement and therapeutic outcomes for individuals with anxiety disorders.

VR provides the opportunity for real-time integration of biofeedback, a process that has historically encountered difficulties in effective execution. By combining VR with physiological monitoring tools, such as heart rate and skin conductance sensors, therapists can offer immediate insights into patients’ stress or anxiety levels during therapy sessions [6]. For instance, a patient with PTSD can learn to manage their stress response by monitoring changes in their heart rate while interacting with a virtual trauma scenario. This prompt feedback encourages the development of self-regulation skills in a way that traditional biofeedback methods cannot achieve. Research by Pallavicini *et al*. [32] has demonstrated the effectiveness of biofeedback-integrated VR in reducing anxiety and improving emotional regulation.

Furthermore, VR introduces innovative techniques such as virtual embodiment and body swapping, which were previously unattainable [6]. For individuals facing body image issues or eating disorders, VR allows them to “experience” a virtual body that reflects a healthier self-image. This immersive, first-person experience can significantly alter their self-perception in ways that conventional verbal or visual methods cannot replicate [5, 7]. Additionally, VR can aid patients dealing with chronic pain by immersing them in virtual environments that distract from physical discomfort, thus reducing their reliance on medication. Studies indicate that virtual embodiment techniques are effective in addressing body image disturbances and chronic pain [33, 34].

One significant advantage of VR is its ability to facilitate avatar-based therapy for individuals suffering from psychosis. Patients experiencing hallucinations or delusions can interact with virtual avatars that represent their symptoms, allowing them to confront and manage these experiences in a tangible way [7]. This method goes beyond traditional verbal communication, providing a more immediate and effective means of symptom management. Research conducted by Craig *et al*. [35] has shown that avatar therapy markedly improves treatment outcomes.

The accessibility and scalability of VR make it an essential tool for reaching patients who may struggle to access traditional treatment. For example, individuals living in remote or underserved areas can participate in VR therapy from the comfort of their homes, thereby reducing barriers to care [6]. This scalability also extends to the training of therapists, who can use VR to enhance their skills in realistic settings without the need for physical resources. A study [36] highlighted VR’s potential to democratize access to mental health care, particularly in resource-limited environments.

## 4. Benefits and Side Effects of Virtual Reality in Mental Health

VR has emerged as a powerful tool in mental health, offering a range of benefits that go beyond the capabilities of traditional therapeutic methods [1]. Key benefits of VR in mental health include the creation of highly controlled, immersive environments, the ability to personalize therapeutic interventions, and the collection of real-time data, which together increase the effectiveness of treatments [36]. This is particularly useful in exposure therapies, where patients can be gradually exposed to stimuli that trigger reactions in a safe and controlled manner. It also allows these exposures to occur without the risks associated with real-world situations, facilitating the treatment of conditions such as phobias and anxiety disorders [37].

Technology allows virtual scenarios to be tailored to the individual needs of each patient, adjusting the level of difficulty and type of stimulus according to the patient’s progress and emotional responses [38]. This is made possible by VR’s ability to monitor real-time data, such as facial expressions and physiological signals, allowing for dynamic adjustments during therapy. In addition, VR facilitates the collection of behavioral and physiological data during therapy sessions, providing valuable insights into the patient’s progress. These data can help therapists to assess the effectiveness of interventions and adjust treatment strategies as needed [39, 40]. The ability to record and analyze these data in real time is a key differentiator that can lead to more accurate and personalized interventions.

Therapy sessions can be delivered both in clinics and at home using VR devices that are becoming increasingly affordable. This not only improves access to treatment but also increases patient engagement and adherence, as they can participate in therapy sessions in an environment that is more comfortable for them [41]. VR also has the potential to be combined with other emerging technologies, such as artificial intelligence (AI) and biofeedback, to create even more effective therapeutic experiences. AI can be used to dynamically adapt virtual environments to the needs of the patient, while biofeedback can provide real-time information about patients’ physiological responses, allowing for immediate adjustments to virtual scenarios [38]. VR offers significant benefits in mental health by providing a safe, controlled and personalized environment for therapeutic interventions. These advances not only improve the effectiveness of treatments, but also expand the possibilities for applying therapy in different contexts and populations, highlighting VR as a promising and innovative tool in mental health.

Although VRET is an innovative and effective approach to treating a variety of mental health disorders, as with any therapeutic intervention, VRET may have side effects that should be considered [6]. One of the most common side effects is physical discomfort, which can include cybersickness, characterized by nausea and dizziness due to the discrepancy between visual experiences and bodily sensations. In addition, prolonged use of VR devices can cause eye strain and visual discomfort [41].

Another aspect to be considered is the temporary increase in anxiety; exposure to situations that provoke fear or anxiety can initially intensify these feelings, leading to an increase in anxiety before the expected decrease occurs [42]. In addition, some people may experience depersonalization and derealization, which are feelings of detachment from themselves or their surroundings, which can be disconcerting [43]. Exposure therapy can also evoke intense emotions and memories of trauma, making it difficult for some patients to cope with these emotional reactions [44]. Another risk is dependence on technology; the ease of access and immersion can lead to overuse, resulting in a reliance on technology to manage anxiety or other emotional issues [45].

Disconnection from reality is another concern, as some individuals may have difficulty reintegrating into the real world, especially if the experience was very intense. Additionally, unwanted physical reactions, such as sweating or rapid heartbeat, may occur, causing discomfort or alarm [45]. Finally, while VRET offers many benefits, it is essential that mental health professionals are aware of these potential adverse effects [46]. Adequate supervision, ongoing assessment, and adaptation of interventions are key to maximizing the benefits and minimizing the risks associated with this therapeutic approach.

## 5. Methodological Challenges and Accessibility of Virtual Reality in Mental Health

The application of VR in mental health faces several methodological and accessibility challenges that need to be addressed to maximize its therapeutic potential. First, a major methodological challenge is the lack of high-quality research. Many studies have used weak methodologies, which prevents conclusive evidence on the efficacy of VR for various mental disorders. There is an urgent need for standardization in VR clinical trials, as there are currently no clear guidelines for such studies [41]. Furthermore, the clinical implementation of VR is hampered by the lack of research investigating the feasibility and efficacy of self-administered treatments, as well as the necessary level of clinician involvement in this process [6]. Another important aspect is the need to conduct pilot tests in diverse communities to assess the cultural appropriateness and acceptability of VR-based mental health interventions [6].

In terms of accessibility, the clinical adoption of VR faces technological, financial, and training barriers that need to be identified and overcome to facilitate its integration into mental health services [41]. Ethical and safety issues are also crucial, especially regarding the integration of AI into VR environments and the safety profile of VR use, with a focus on adverse events [6].

Technical issues related to VR, such as screen size, resolution, field of view, type of device used, immersive system, and sense of presence are key to improving clinical outcomes and should be carefully considered. Furthermore, the interaction of VR with other services, such as telemedicine, also has significant potential to improve therapeutic outcomes [41]. Therefore, it is essential that there is a greater focus on both the development and clinical validation of VR, which could significantly improve the quality of clinical research in VR and subsequently have a positive impact on the field of mental health [6].

## 6. Future Perspectives for Virtual Reality Research in Mental Health

VR represents a promising frontier in psychiatry, but its potential must be fully realized by addressing its current challenges. Future research should focus on large-scale studies with methodological rigor to validate the efficacy of VR interventions [6]. Furthermore, it is crucial to investigate the underlying mechanisms by which VR exerts its therapeutic effects, which could inform the personalization of interventions [1].

The integration of VR with AI and biofeedback represents a groundbreaking frontier in mental health care, offering opportunities to create highly personalized and adaptive therapeutic experiences [6]. In particular, AI plays a crucial role in enabling the analysis of large volumes of data generated during VR sessions, such as eye movements, facial expressions, and physiological signals, to detect patterns and predict emotional responses [37]. The use of AI in VR enables the creation of dynamic virtual environments that adapt in real time to the patient’s needs and responses [6]. For example, AI-controlled virtual characters can interact with patients in a natural and empathetic way, offering feedback and emotional support during treatment. This not only increases patient engagement but also allows for a high degree of personalization in therapy, adjusting the content and difficulty of tasks according to the patient’s program [6].

Biofeedback, on the other hand, involves the use of devices that monitor physiological signals, such as heart rate and brain activity, to provide real-time feedback to the patient and therapist [42]. These data can be used to automatically adjust the VR environment, providing a level of personalization that is difficult to achieve with traditional methods. For example, if a patient shows signs of increased anxiety, the environment can be modified to introduce calming elements or to decrease the intensity of the stimulus [47]. The combination of VR, AI, and biofeedback can therefore facilitate a therapeutic approach that is both responsive and proactive. This means that therapy not only reacts to the patient’s current conditions but also adapts in ways that anticipate and mitigate adverse emotional responses, promoting more effective and engaging treatment [6]. These integrated technologies have the potential to revolutionize the treatment of a wide range of mental health conditions, increasing the effectiveness of interventions and making them more accessible. As research progresses, these approaches are expected to become an integral part of standard therapeutic practices, offering new ways to address complex problems such as anxiety, depression, and stress disorders [6].

As VR becomes more integrated into clinical practice, ethical and practical issues arise that must be considered. Training mental health professionals in the effective and ethical use of VR is essential to ensure that the technology is used responsibly. Clinicians should be aware of potential limitations and risks associated with VR, as well as best practices for its implementation.

## 7. Conclusion

VR offers a new dimension to the treatment of psychiatric disorders, with the potential to transform the way interventions are delivered. However, for VR to reach its full potential in psychiatry, methodological challenges must be overcome, accessibility must be expanded, and research must continue to better understand its effects. With continued investment in research and development, VR could become a standard tool in the psychiatric therapeutic toolbox, offering new possibilities for patients and healthcare professionals. Successful implementation in psychiatry requires a collaborative effort by researchers, clinicians, and technology developers to ensure that interventions are safe, effective, and accessible.
